# Effect of “one disease, one product” combined with self-efficacy nursing on cardiac function and quality of life in patients undergoing rheumatic heart valve replacement

**DOI:** 10.1097/MD.0000000000043450

**Published:** 2025-08-01

**Authors:** Qin Fan, Haiyan Chen, Birun Huang, Xiulan Zhang

**Affiliations:** a Department of Vascular Surgery, The First Affiliated Hospital of Yangtze University, The First People’s Hospital of Jingzhou, Jingzhou City, Hubei, China; b Department of Radiology, The First Affiliated Hospital of Yangtze University, The First People’s Hospital of Jingzhou, Jingzhou City, Hubei, China.

**Keywords:** rheumatic heart disease, self-efficacy, valve replacement

## Abstract

Rheumatic heart disease (RHD) is a chronic condition that frequently necessitates valve replacement surgery. However, the postoperative recovery period can be prolonged, and persistent cardiac dysfunction may occur. This underscores the need for effective nursing interventions to improve patient outcomes. This study aimed to evaluate the effectiveness of the “one disease, one product” nursing model combined with self-efficacy enhancement on cardiac function and quality of life in patients undergoing RHD valve replacement. A retrospective analysis was conducted on 100 patients who underwent RHD valve replacement between January and June 2022. Based on nursing history, patients were assigned to an experimental group (n = 50) or a control group (n = 50). The experimental group received “one disease, one product” nursing combined with self-efficacy interventions, while the control group received standard postoperative care. Cardiac function, complication rates, quality of life, and nursing satisfaction were assessed and compared between groups before and after the intervention. Patients in the experimental group showed significantly greater improvements in left ventricular ejection fraction, cardiac output, and left ventricular end-diastolic diameter compared to the control group (*P* < .001). The incidence of complications was also lower in the experimental group. Quality of life measures—including medical management, physical strength, social functioning, daily activities, and work performance—were significantly higher in the experimental group (*P* < .05). Overall nursing satisfaction in the experimental group reached 90.00%, significantly exceeding that of the control group (*P* = .026). The combination of the “one disease, one product” care model with self-efficacy enhancement significantly improves cardiac function, quality of life, and nursing satisfaction in patients undergoing valve replacement for RHD. This integrated nursing approach holds considerable promise for improving postoperative recovery and patient-centered outcomes in similar clinical settings.

## 1. Introduction

Rheumatic heart disorder (RHD) is a progressive condition characterized by damage to the heart valves resulting from recurrent rheumatic fever, most commonly affecting the mitral and aortic valves. In severe cases, this leads to valvular stenosis or regurgitation, which may ultimately progress to heart failure if not treated promptly.^[1–3]^ For patients with advanced RHD where medical and interventional therapies are ineffective, valve replacement surgery remains the standard treatment.^[4,5]^ However, the postoperative recovery process is often prolonged, complicated by residual or recurrent cardiac dysfunction, anticoagulation-related risks, and challenges in lifestyle adjustment.^[6,7]^ These issues highlight the urgent need for effective, structured nursing interventions that not only support physiological recovery but also promote long-term physical, emotional, and social well-being.

In response to the demand for individualized care in chronic disease management, the “one disease, one product” nursing model has emerged as a promising framework. This approach involves developing tailored nursing interventions and educational materials specific to each disease, thereby promoting patient-centered care and improving adherence through practical tools, psychosocial support, and behavior modification strategies.^[8,9]^ Given the chronic course of RHD and the high probability of complications after surgery, such a personalized and standardized model is particularly suitable for this population.^[10,11]^

In parallel, the concept of self-efficacy-based nursing—grounded in Bandura theory—has gained recognition for enhancing patients’ confidence and ability to manage their health conditions. Studies have shown that patients with higher self-efficacy demonstrate better compliance, rehabilitation participation, and psychological adjustment, which contribute to improved outcomes in cardiac surgery and chronic cardiovascular conditions.^[12]^ Furthermore, the integration of self-efficacy support into nursing care has been shown to reduce hospital readmission, improve cardiac function, and enhance quality of life.^[13,14]^

Although both the “one disease, one product” model and self-efficacy-based nursing have shown effectiveness independently, few studies have examined the combined impact of these 2 strategies in postoperative management of RHD patients following valve replacement. This study seeks to address this gap by evaluating the clinical effects of combining these 2 models—personalized structured nursing + self-efficacy enhancement—on cardiac function, quality of life, and patient satisfaction in RHD patients post-surgery.

We hypothesize that this combined intervention will result in significantly better functional recovery, fewer complications, and higher levels of patient satisfaction compared to standard care. The findings of this study aim to provide practical implications for the design of comprehensive, patient-centered postoperative nursing programs in cardiovascular care settings.

## 2. Materials and methods

### 2.1. Study design

This study was approved by the Ethics Committee of the First Affiliated Hospital of Yangtze University. In this trial, we used a retrospective cohort design that enrolled 100 cases of RHD in our hospital between January 2022 and June 2024. The patients were divided into experimental group (n = 50) and control group (n = 50). Patients in test group were given “one disease, one product” and self-efficacy measures, while those in the control group were given standard treatment.Inclusion criteria were: (1) diagnosis of RHD confirmed by angiography and X-ray; (2) completion of valve replacement surgery; (3) signed informed consent. Exclusion criteria were: (1) liver or kidney disease; (2) cognitive or mental disorders; (3) pregnancy or lactation; (4) malignant tumors.

### 2.2. Grouping

This study employed a single-blind design in which patients were unaware of their group allocation to minimize potential psychological bias and expectancy effects. All nursing interventions in both groups were carried out by qualified nursing staff trained under standardized protocols. The clinical procedures strictly adhered to hospital operating standards and national nursing guidelines. Routine audits and supervision ensured the fidelity of intervention delivery across both groups, and no deviation or reduction in standard care was permitted in the control group.

#### 2.2.1. Control group

Patients in the control group were treated with the standard care, which consisted of surgery education, vital sign monitoring, dietary and lifestyle guidance, pain management, and follow-up after discharge. Care involved explaining the surgical procedure and precautions in detail to ensure patients and families fully understood the treatment. Vital signs like heartbeat and blood pressure were carefully monitored so that they could be treated immediately.Patients were given dietary advice recommending a low-salt, low-fat, easily digestible diet, along with lifestyle adjustments to encourage moderate activity for recovery. Pain management was provided as needed to ensure postoperative comfort, and a follow-up plan was established for continued individualized care after discharge.

#### 2.2.2. Experimental group

The experimental group received the “one disease, one product” nursing model combined with self-efficacy measures. A team of 5 dedicated nurses tailored individualized care plans based on each patient’s condition and understanding of the disease. Rehabilitation guidance included in-bed limb exercises and standing/walking training, with daily progress recorded and personalized plans developed. Anticoagulation management involved a detailed “Anticoagulation Record Form” that documented patient information, surgery date, and medication details to monitor drug use and effectiveness daily. Pain management was administered as needed, with effectiveness promptly evaluated. Discharge instructions covered medication reminders, low-fat, low-salt dietary recommendations, and postoperative exercise plans to ensure a safe and effective recovery, supplemented by weekly SMS reminders for postoperative care and follow-up.

Self-efficacy measures included training the nursing team to enhance professional skills, assessing patient emotions, providing one-on-one psychological support, and involving psychologists for motivational language. Patients were encouraged through examples of those who demonstrated high compliance and good recovery. Stage-specific goals were established based on each patient’s condition and habits, with ongoing encouragement and support provided to improve self-efficacy, foster positive attitudes, and promote rehabilitation.

### 2.3. Outcome measures

This retrospective study collected data through the nursing system and questionnaires. This retrospective study collected data through the hospital’s nursing system and structured questionnaires to evaluate treatment outcomes. Cardiac function was assessed using objective indicators, including left ventricular ejection fraction (LVEF), cardiac output (CO), and left ventricular end-diastolic diameter (LVEDD), with comparisons made before and after the intervention. All echocardiographic measurements were performed by technicians who were blinded to the group allocation to reduce observer bias.

Quality of life was evaluated using the Chinese Cardiovascular Quality of Life Questionnaire, covering domains such as physical health, disease status, social functioning, daily activities, and work performance. To minimize bias, questionnaire analysis was conducted by independent researchers who were not involved in the intervention delivery and were blinded to group assignments.

Nursing satisfaction was measured using a customized satisfaction survey, which included items on service attitude, professional skills, and the ward environment. Participants completed the survey anonymously to encourage honest feedback and reduce social desirability bias.

### 2.4. Data analysis

Using SPSS 25.0 to analyze the data. Continuous variables, such as LVEF, CO, LVEDD, and quality of life scores, the values of the mean ± SD were measured with an independent sample *t* test. Classified variables were analyzed using chi-square analysis method. Below 0.05 was regarded as a statistical significance.

## 3. Results

### 3.1. Comparison of baseline data between groups

In this paper, the population and clinic features of test and control were compared. The results showed that there were no statistically significant differences with respect to age, sex, duration of illness, New York Heart Association function classification, and surgical treatment (all *P* > .05). Since baseline data showed no significant differences, it can be inferred that differences in follow-up outcomes are primarily attributable to the nursing measures rather than baseline variations, enhancing the effectiveness and reliability of the findings of the study (Table 1).

**Table 1:** Comparison of general baseline data between the 2 groups.

Variables	Experimental group (n = 50)	Control group (n = 50)	*P* value
Age (year, mean ± SD)	50.32 ± 5.32	51.02 ± 5.77	.591
Gender, n (%)			
Male	26 (52.00)	28 (56.00)	.820
Female	24 (48.00)	22 (44.00)	
Course of disease (year, mean ± SD)	5.52 ± 1.32	5.47 ± 1.35	.654
NYHA functional classification, n (%)			.693
Class II	12 (24.00)	10 (20.00)	
Class III	27 (54.00)	28 (56.00)	
Class IV	11 (22.00)	12 (24.00)	
Surgical type, n (%)			.457
Mitral valve replacement	28 (56.00)	30 (60.00)	
Aortic valve replacement	22 (44.00)	20 (40.00)	

Consecutive variables (age, duration of illness) were compared with an independent *t* test, while categorical variables (gender, NYHA classification, and surgical type) were compared using a chi-square test.

NYHA = New York Heart Association.

### 3.2. Cardiac function

Before treatment, LVEF, CO, and LVEDD did not differ significantly between experimental and control groups (all *P* > .05), indicating comparable baseline conditions. After treatment, all cardiac functional parameters were significantly improved in the test group. The LVEF of experiment group was raised from 40.49 ± 3. 73 to 55.24 ± 4. 86, compared to an increase from 39.22 ± 3.95 to 49.28 ± 4.49 in the control group (*P* < .001). The experimental group also showed a significant increase in CO to 4.61 ± 0.35, which was higher than the control group (*P* < .001). Additionally, LVEDD in the experimental group increased significantly to 77.59 ± 5.82, compared to 70.43 ± 4.93 in the control group, with a statistically significant difference (*P* < .001). Overall, the “one disease, one product” nursing combined with self-efficacy was more effective in improving cardiac function, cardiac output, and left ventricular diastolic function compared to standard care (Table 2).

**Table 2:** Comparison of heart function between 2 groups.

Variables	Time	Experimental group (n = 50)	Control group (n = 50)	*t*	*P*
LVEF (%)	Before treatment	40.49 ± 3.73	39.22 ± 3.95	0.040	.968
	After treatment	55.24 ± 4.86	49.28 ± 4.49	6.463	<.001
CO (L)	Before treatment	3.31 ± 0.28	3.33 ± 0.27	0.032	.975
	After treatment	4.61 ± 0.35	4.08 ± 0.32	7.702	<.001
LVEDD (mm)	Before treatment	62.94 ± 5.52	64.45 ± 5.36	0.054	.968
	After treatment	77.59 ± 5.82	70.43 ± 4.93	5.710	<.001

### 3.3. Complications

**Figure 1.**
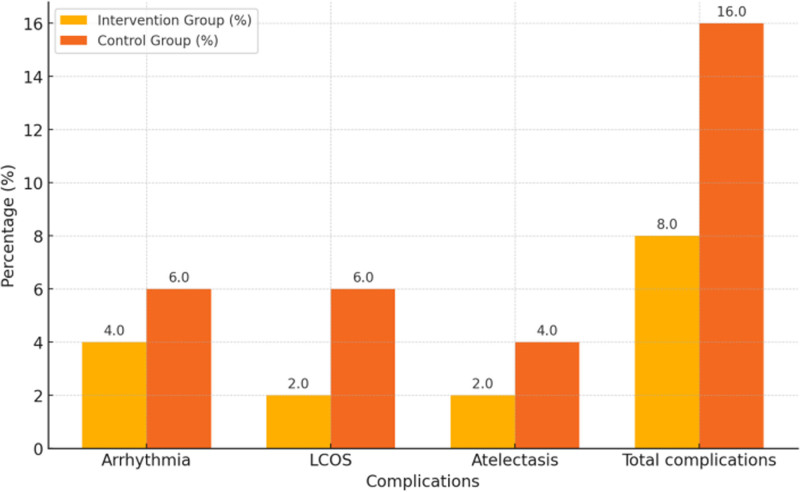
Comparison of complications between the 2 groups.

The comparison of complication rates between the 2 groups showed that the overall complication rate in the experimental group was 8.00% (4/50), compared to 16.00% (8/50) in the control group, with a statistically significant difference (*P* = .036). Specifically, the incidence of arrhythmia in the experimental group was 4.00% (2/50), low cardiac output syndrome was 2.00% (1/50), and atelectasis was 2.00% (1/50). In contrast, the corresponding rates in the control group were 6.00% (3/50), 6.00% (3/50), and 4.00% (2/50), respectively. Therefore, the incidence of all complications in the test group was lower than in the control group (Table 3 and Fig. 1).

**Table 3:** Comparison of complications between the 2 groups.

Variables	Experimental group (n = 50)	Control group (n = 50)	*χ* ^2^	*P* value
Total complications, n (%)	4 (8.00)	8 (16.00)	4.210	.036
Arrhythmia, n (%)	2 (4.00)	3 (6.00)		
LCOS, n (%)	1 (2.00)	3 (6.00)		
Atelectasis, n (%)	1 (2.00)	2 (4.00)		

LCOS = low cardiac output syndrome.

### 3.4. Quality of life

Before treatment, there was no statistically significant difference in quality of life indicators (e.g., medical care, disease status, physical strength, social function, daily activities, and work status) (all *P* > .05), indicating comparable baseline conditions. After treatment, all measured indicators showed significant improvement. The health care score of test group was raised from 73.04 to 81.36, and that of the control group was raised from 72.75 to 77.63 (*P* < .001). The disease status score in the experimental group rose from 71.81 to 84.59, compared to 79.65 in the control group (*P* = .001). Physical strength and work status scores also improved significantly, with the work status score rising from 58.32 to 81.25 (*P* < .001). The experimental group showed significant increases in social function and overall quality of life scores, reaching 86.58 and 85.37, respectively, compared to the control group (*P* < .05). These results indicate that “one disease, one product” nursing combined with self-efficacy significantly improved patients’ quality of life (Table 4).

**Table 4:** Comparison of 2 groups’ quality of life scores.

Variables	Time	Experimental group (n = 50)	Control group (n = 50)	*t*	*P*
Medical treatment	Before treatment	73.04 ± 4.25	72.75 ± 5.14	0.371	.735
	After treatment	81.36 ± 5.21	77.63 ± 5.55	3.652	<.001
State of illness	Before treatment	71.81 ± 4.62	70.09 ± 4.50	0.523	.514
	After treatment	84.59 ± 5.36	79.65 ± 5.75	3.658	.001
Physical power	Before treatment	60.32 ± 6.79	61.07 ± 5.37	1.682	.141
	After treatment	71.25 ± 5.44	65.24 ± 3.34	4.142	<.001
Social function	Before treatment	72.12 ± 4.61	72.79 ± 4.33	0.430	.829
	After treatment	86.58 ± 5.75	80.81 ± 5.23	2.947	.002
General living	Before treatment	70.61 ± 4.51	70.01 ± 4.32	0.296	.845
	After treatment	85.37 ± 5.13	82.32 ± 5.34	3.530	.001
Work status	Before treatment	58.32 ± 6.89	61.07 ± 5.47	1.482	.151
	After treatment	81.25 ± 5.24	74.24 ± 3.54	4.132	<.001

### 3.5. Nursing satisfaction

No statistical significance was found on the level of “satisfactory,” “moderate satisfaction” and “highly satisfactory” among the 2 groups (all *P* > .05), indicating similar experiences in these specific aspects. But the total degree of satisfaction in the experiment group was much better than that in the control group, and the satisfactory rate was 96. 00% (*P* = .026). This suggests that “one disease, one product” nursing combined with self-efficacy significantly improved overall satisfaction, likely due to the more personalized and targeted care provided in the experimental group, better meeting patients’ needs (Table 5).

**Table 5:** Comparison of nursing satisfaction between 2 groups.

Variables	Experimental group (n = 50)	Control group (n = 50)	*χ* ^2^	*P*
Dissatisfied, n (%)	2 (4.00)	4 (8.00)	2.561	.072
Quite satisfied, n (%)	22 (44.00)	18 (36.00)	0.560	.469
Very satisfied, n (%)	26 (52.00)	28 (56.00)	0.681	.628
Overall satisfaction, n (%)	48 (96.00)	46 (80.00)	4.214	.026

## 4. Discussion

The main findings of this study demonstrate that the “one disease, one product” nursing model combined with self-efficacy enhancement significantly improved cardiac function, reduced postoperative complications, and enhanced both quality of life and nursing satisfaction in patients with RHD undergoing valve replacement. Patients in the experimental group exhibited greater improvements in key cardiac parameters—including LVEF, CO, and LVEDD—compared to those receiving standard care. In addition, quality of life scores related to physical strength, social functioning, and work ability were markedly higher in the intervention group. The elevated satisfaction levels further support the effectiveness of combining individualized nursing with self-efficacy enhancement strategies.

These results align with prior studies in the field of postoperative care for patients with coronary artery disease, while also presenting several distinctive findings. For instance, the LVEF in the experimental group increased significantly to 55.24 ± 4.86, exceeding the 47.3 ± 5.2 improvement reported under traditional care by Oldridge et al.^[15]^ Similarly, compared to the findings of Spertus et al,^[16]^ our approach yielded greater enhancements in LVEF, likely due to its patient-centered and personalized nursing approach. Regarding quality of life, the most notable improvement was in work status, which rose from 58.32 to 81.25, consistent with the trends observed by Ball et al.^[17]^ However, our relatively short follow-up period—compared to the multicenter longitudinal study by Zühlke et al^[10]^—may limit the assessment of long-term effects. Nursing satisfaction in our study reached 96.00%, significantly higher than the 82.5% reported by Riegel et al,^[18]^ suggesting that integrating individualized care with motivational support may more effectively meet patient needs.

A distinctive strength of this study lies in its novel application of the “one disease, one product” model in combination with self-efficacy enhancement for postoperative RHD patients—a population where such dual strategies have rarely been studied. In contrast to conventional nursing approaches, this combined model addresses both the physical and psychological dimensions of recovery.^[19,20]^ Notably, the observed LVEF improvement of 14.75 percentage points in the experimental group surpasses outcomes reported in previous literature,^[21]^ likely reflecting the emphasis on patient participation and personalized rehabilitation planning.^[22]^ Moreover, the quality of life assessment in this study extended beyond physiological recovery to include indicators such as social engagement and work reintegration.^[23,24]^ For example, the social functioning score in the experimental group reached 86.58—an outcome superior to those reported in prior studies,^[25]^ possibly attributable to our targeted efforts to enhance patients’ social support networks. Importantly, our study also innovatively explored the relationship between nursing satisfaction and clinical outcomes, uncovering a meaningful positive correlation that may offer new perspectives on evaluating nursing care quality.

Despite the promising results, this study has several limitations that should be acknowledged. First, the sample size was relatively small, and the study was conducted at a single center, which may limit the generalizability of the findings. Second, although the baseline characteristics were balanced between groups, we did not explicitly control for potential confounding variables such as socioeconomic status, educational background, underlying comorbidities, or levels of family support. These factors may have influenced patients’ adherence, recovery rates, or perceptions of care. Third, due to the practical nature of the nursing interventions, a double-blind design was not feasible. While a single-blind approach was employed—where patients were unaware of group allocation—and evaluators were blinded to reduce bias, the possibility of placebo effects or expectancy bias cannot be completely ruled out, particularly for subjective measures such as satisfaction and quality of life. Additionally, the extra attention received by the experimental group may have enhanced their motivation, introducing a performance bias. Lastly, part of the outcome data relied on self-reported questionnaires, which may be affected by observer or response bias, despite efforts to standardize and anonymize data collection. Future studies should consider larger, multicenter randomized controlled trials with stratified sampling to better account for demographic and psychosocial variability. The use of more objective clinical endpoints and long-term follow-up is also recommended. Furthermore, integrating digital tools such as telehealth platforms may improve intervention consistency and reduce bias related to caregiver interaction intensity.

## 5. Conclusions

In conclusion, the “one disease, one product” nursing model combined with self-efficacy enhancement significantly improved cardiac function, quality of life, and nursing satisfaction in patients with rheumatic heart disease undergoing valve replacement, demonstrating the importance of personalized care and self-management in postoperative rehabilitation. This approach has distinct advantages in enhancing left ventricular ejection fraction, cardiac output, and overall quality of life. Therefore, it is recommended for widespread use in clinical nursing to promote better postoperative outcomes and increased patient satisfaction. Future research should focus on optimizing this nursing strategy and validating its effectiveness across various medical settings through multicenter, large-scale, and long-term follow-up studies, benefiting a broader patient population.

## Author contributions

**Conceptualization:** Qin Fan, Haiyan Chen, Birun Huang, Xiulan Zhang.

**Formal analysis:** Qin Fan, Haiyan Chen, Birun Huang, Xiulan Zhang.

**Funding acquisition:** Qin Fan, Haiyan Chen, Birun Huang, Xiulan Zhang.

**Investigation:** Qin Fan, Haiyan Chen, Birun Huang, Xiulan Zhang.

**Methodology:** Qin Fan, Haiyan Chen, Birun Huang, Xiulan Zhang.

**Supervision:** Birun Huang, Xiulan Zhang.

**Validation:** Birun Huang, Xiulan Zhang.

**Writing – original draft:** Qin Fan, Haiyan Chen, Birun Huang, Xiulan Zhang.

**Writing – review & editing:** Qin Fan, Haiyan Chen, Birun Huang, Xiulan Zhang.
